# Neutrophil-mediated hypoxia drives pathogenic CD8^+^ T cell responses in cutaneous leishmaniasis

**DOI:** 10.1172/JCI177992

**Published:** 2024-06-04

**Authors:** Erin A. Fowler, Camila Farias Amorim, Klauss Mostacada, Allison Yan, Laís Amorim Sacramento, Rae A. Stanco, Emily D.S. Hales, Aditi Varkey, Wenjing Zong, Gary D. Wu, Camila I. de Oliveira, Patrick L. Collins, Fernanda O. Novais

**Affiliations:** 1 Department of Microbial Infection and Immunity, College of Medicine, The Ohio State University, Columbus, Ohio, USA.; 2 Department of Pathobiology, School of Veterinary Medicine and; 3 Division of Gastroenterology and Hepatology, Perelman School of Medicine, University of Pennsylvania, Philadelphia, Pennsylvania, USA.; 4 Instituto Gonçalo Moniz, FIOCRUZ, Salvador, Brazil.; 5 Instituto Nacional de Ciência e Tecnologia em Doenças Tropicais, Salvador, Brazil.

**Keywords:** Infectious disease, Inflammation, Hypoxia, Neutrophils, T cells

## Abstract

Cutaneous leishmaniasis caused by *Leishmania* parasites exhibits a wide range of clinical manifestations. Although parasites influence disease severity, cytolytic CD8^+^ T cell responses mediate disease. Although these responses originate in the lymph node, we found that expression of the cytolytic effector molecule granzyme B was restricted to lesional CD8^+^ T cells in *Leishmania*-infected mice, suggesting that local cues within inflamed skin induced cytolytic function. Expression of Blimp-1 (*Prdm1*), a transcription factor necessary for cytolytic CD8^+^ T cell differentiation, was driven by hypoxia within the inflamed skin. Hypoxia was further enhanced by the recruitment of neutrophils that consumed oxygen to produce ROS and ultimately increased the hypoxic state and granzyme B expression in CD8^+^ T cells. Importantly, lesions from patients with cutaneous leishmaniasis exhibited hypoxia transcription signatures that correlated with the presence of neutrophils. Thus, targeting hypoxia-driven signals that support local differentiation of cytolytic CD8^+^ T cells may improve the prognosis for patients with cutaneous leishmaniasis, as well as for other inflammatory skin diseases in which cytolytic CD8^+^ T cells contribute to pathogenesis.

## Abstract 2

Neutrophils recruited by *Leishmania* infection consume tissue oxygen to produce ROS, leading to hypoxia, increased cytotoxicity in CD8^+^ T cells, and damaged tissue.

## Graphical abstract

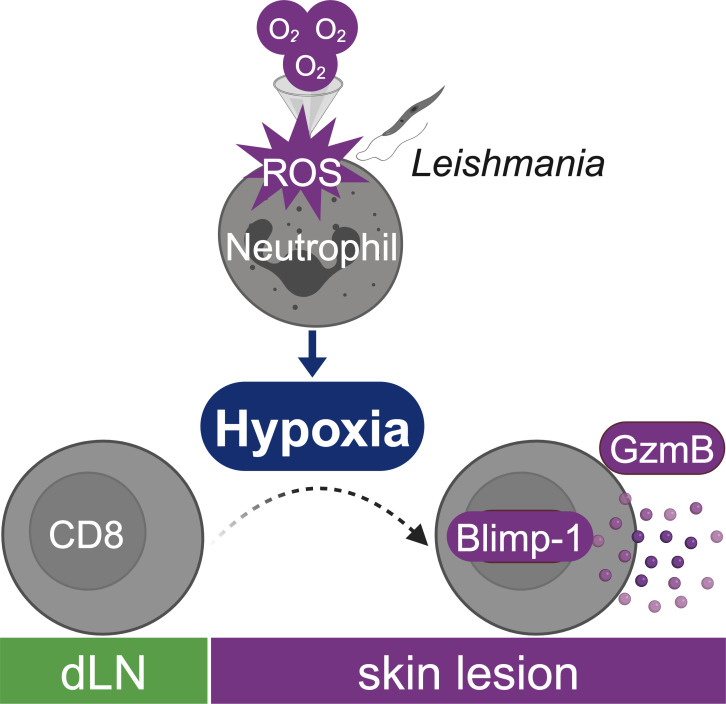

## Introduction

Cutaneous leishmaniasis is caused by *Leishmania* parasites and exhibits a wide range of clinical manifestations, from self-healing lesions to chronic, debilitating infections (1). No vaccines exist for leishmaniasis, and antiparasitic drugs are often ineffective (2). Although the parasites influence disease severity, immune responses are often the major driver of disease. For example, CD8^+^ T cells in lesions are recruited to target parasite-infected cells and kill infected host cells through granule-mediated cytotoxicity. However, the destruction of infected cells does not kill *Leishmania* but leads to the release of parasites that metastasize to distant cutaneous sites (3). In addition, cell death induced by CD8^+^ T cells activates the NLRP3 inflammasome, IL-1β release, and chronic neutrophil recruitment and enhances inflammation (3–6). The importance of this pathway is supported by our previous analysis of lesions from patients in whom the expression of granzyme B (*GZMB*), perforin 1 (*PRF1*), and *IL1B* was associated with treatment failure (7). While murine and human studies underscore the pathogenic consequences of cytotoxicity, the microenvironmental cues and downstream mechanisms that drive cytolytic programs in lesional CD8^+^ T cells remain unknown.

In response to *Leishmania* infection, CD8^+^ T cells are activated and expand in draining lymph nodes (dLNs). The activated cells then travel to inflammatory sites, where they kill infected cells. Although infections also induce the differentiation of effector CD4^+^ T cells in secondary lymphoid organs, CD8^+^ T cells with a cytotoxic effector function are generally absent in LNs (8–11). A prior study implicated programmed cell death 1 (PD-1) ligation on dLN CD8^+^ T cells in suppressing their expression of GzmB, a process that is likely advantageous, since it would limit the killing of antigen-presenting cells (11). While such findings suggest that CD8^+^ T cell cytolytic effector function is only triggered once CD8^+^ T cells enter inflammatory sites (12, 13), the local cues that drive this response remain unclear. As the CD8^+^ T cell cytolytic program can have both positive and negative consequences for the host (14–33), understanding the mechanisms that drive cytotoxic CD8^+^ T cell programs remains an important goal for unraveling the pathogenesis of numerous diseases such as alopecia, vitiligo, bullous pemphigoid, Stevens-Johnson syndrome, and toxic epidermal necrolysis, in which cytolytic CD8^+^ T cells contribute to pathogenesis (15, 32, 34–37).

One potential mechanism relevant to inflamed tissues is hypoxia, which occurs when O_2_ consumption by infiltrating cells exceeds the supply (38) and differentially affects immune cell types. In *Leishmania* infection, hypoxia has been studied exclusively in macrophages and DCs (39, 40), where it can either promote or block effector responses, depending on the parasite species and the cell type (41–49). Here, we found that hypoxia within *Leishmania*-infected lesions directly promoted cytolytic effector function in newly recruited CD8^+^ T cells. Mechanistically, we identified a feed-forward loop, initiating with early recruitment of neutrophils that consume O_2_ to produce ROS, which promoted a persistently hypoxic environment, elevated the number of cytolytic CD8^+^ T cells, perpetuated neutrophil recruitment (4), and exacerbated tissue damage. Thus, our findings indicate that targeting cytolytic CD8^+^ T cells driven by hypoxic microenvironments will improve the prognosis for patients with cutaneous leishmaniasis and other inflammatory skin diseases.

## Results

### CD8^+^ T cells express GzmB in leishmanial lesions but not in the dLNs.

**Figure 1:**
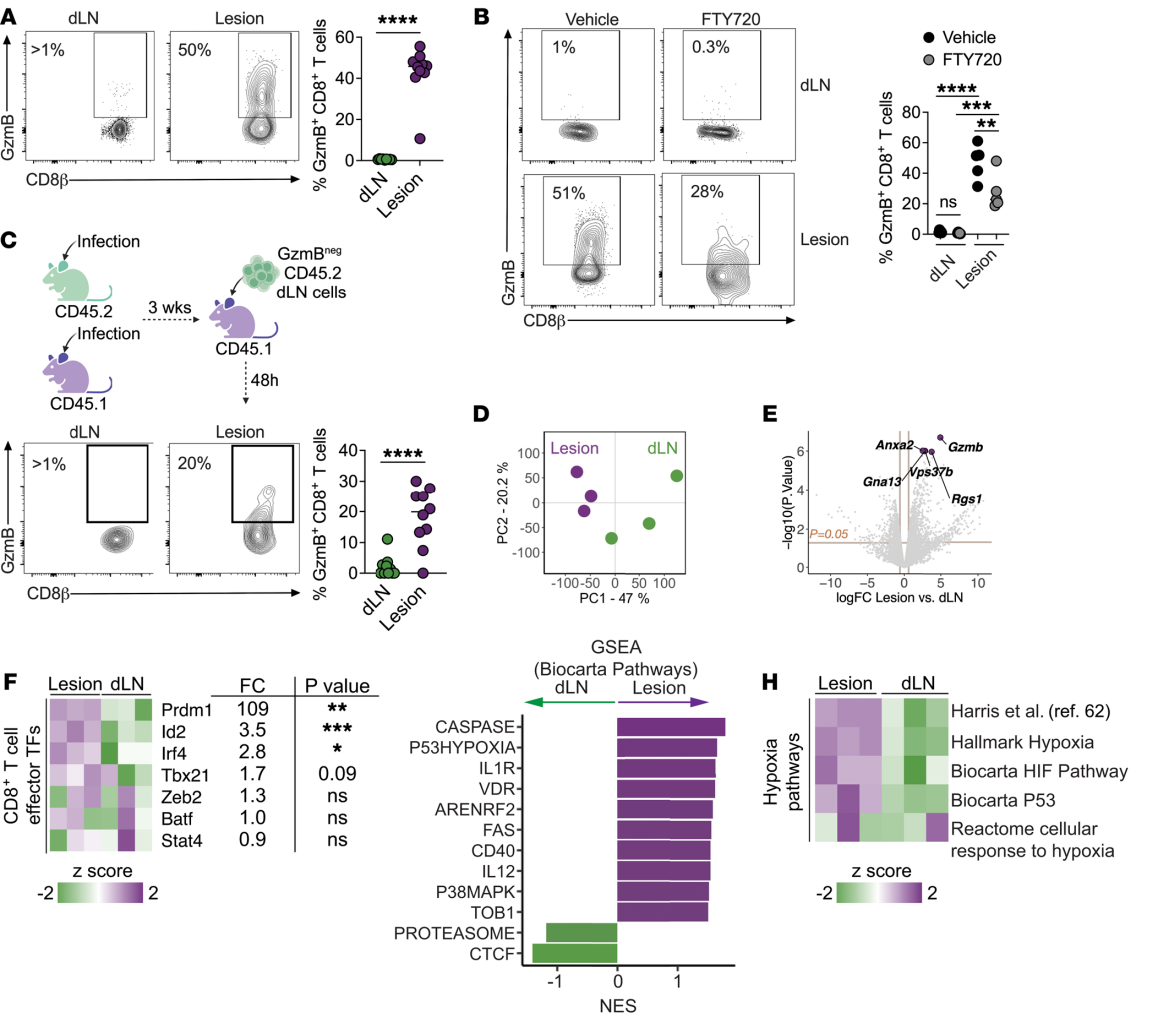
CD8^+^ T cell function is tissue specific. (**A**) GzmB expression in CD8^+^ T cells from dLNs and lesions from C57BL/6 mice in week 2 after infection with *L*. *major*. Data are representative of more than 3 experiments with at least 4 mice per experiment. (**B**) GzmB expression in CD8^+^ T cells from dLNs and lesions from *L*. *major–*infected C57BL/6 mice treated with FTY720 or vehicle daily for 10 days, 12 days after infection. Data are from 2 independent experiments with 5 mice per group. (**C**) Schematic representation of the transfer of purified CD8^+^ T cells from dLNs of CD45.2 mice into the lesions of CD45.1 recipients, both infected for 3 weeks. Flow cytometric plot shows GzmB expression in donor CD8^+^ T cells from dLNs and lesions from recipient mice 48 hours after transfer. Data were combined from 2 independent experiments. (**D**–**H**) RNA-Seq analyses of antigen-experienced (CD44^hi^) CD8^+^ T cells purified from dLNs and lesions of *L*. *major*–infected mice in week 5. (**D**) PCA showing PC1 and PC2. (**E**) Volcano plot highlighting the top 5 DEGs. The orange line indicates an adjusted *P* value of 0.05. (**F**) Heatmap showing the expression of genes encoding transcription factors (TFs) associated with effector-like T cell functions and the FC between lesional and dLN CD8^+^ T cells. (**G**) GSEA (Biocarta) showing pathways enriched in dLNs (green) and lesions (purple). NES, normalized enrichment score. (**H**) Heatmap of hypoxia-related pathways enriched in lesional and dLN CD8^+^ T cells. *P ≤ 0.05, **P ≤ 0.01, ***P ≤ 0.001, and ****P ≤ 0.0001, by 2-tailed Student’s *t* test (**A** , **C**, and **F**) and 1-way ANOVA (**B**).

Cytotoxic CD8 T cells are pathogenic in cutaneous leishmaniasis (3, 4, 6, 7, 50–54), but their anatomical distribution and mechanisms that regulate their induction remain unclear. In this regard, parasites are found in both dLNs and skin lesions. To determine whether CD8^+^ T cell cytotoxic effector function is present in both tissues, we assessed the expression of the cytotoxic effector molecule GzmB. C57BL/6 mice were infected with *Leishmania* in the ear, and 2 weeks after infection, the frequency of GzmB^pos^ antigen-experienced (CD44^hi^) CD8^+^ T cells was assessed by flow cytometry. While GzmB-expressing CD8^+^ T cells were abundant in the infected skin, GzmB expression was nearly absent in the dLNs (Figure 1A). To test whether other effector functions by CD8^+^ T cells are intact in dLNs, we looked for IFN-γ production, which is required for protection against the parasite (1). As demonstrated previously (55), we detected IFN-γ^pos^ CD8^+^ T cells in dLNs, whereas IFN-γ expression was diminished in lesions (Supplemental Figure 1A; supplemental material available online with this article; https://doi.org/10.1172/JCI177992DS1). Collectively, these data demonstrate a tissue-specific effector response, with CD8^+^ T cells in dLNs exhibiting a protective phenotype through the production of IFN-γ and CD8^+^ T cells in lesions exhibiting a pathogenic phenotype by expressing GzmB. Since it is well described that cytotoxic CD8^+^ T cells are pathogenic in cutaneous leishmaniasis (3–7, 50, 51, 56, 57), we sought to determine how CD8^+^ T cells become cytotoxic. First, we asked if GzmB^pos^ CD8^+^ T cells were preferentially recruited to the skin. To address this question, we treated mice with FTY720, a sphingosine 1-phosphate receptor agonist that blocks the egress of T cells from the dLNs. As expected, we observed a defect in the recruitment of T cells to the lesions of FTY720-treated mice compared with vehicle control–treated mice, as observed by a significant decrease in the frequency of CD3^pos^ cells in lesions (Supplemental Figure 1B). Although FTY720-treated mice had fewer CD8^+^ T cells in lesions, those CD8^+^ T cells recruited to the inflamed skin still expressed GzmB (Figure 1B). If GzmB^pos^ CD8^+^ T cells are preferentially recruited to cutaneous lesions, GzmB^pos^ CD8^+^ T cells should accumulate in the dLNs of FTY720-treated mice. We found no differences in the frequency of GzmB^pos^ CD8^+^ T cells in the dLNs of mice treated with FTY720 or vehicle (Figure 1B), suggesting that rather than preferential recruitment, exposure to the lesion microenvironment induced a cytotoxic profile in CD8^+^ T cells. To directly test this, we infected C57BL/6 CD45.1 or CD45.2 congenic mice with *Leishmania* and purified CD8^+^ T cells from dLNs of CD45.2 donor mice (GzmB^neg^) 3 weeks after infection. Donor dLN CD8^+^ T cells were then transferred directly into the lesions of infected CD45.1 animals (Figure 1C, schematic). Notably, we found that GzmB^neg^ CD8^+^ T cells became GzmB-expressing CD8^+^ T cells in the lesions (Figure 1C). In contrast, CD8^+^ T cells that migrated back to dLNs remained GzmB^neg^ (Figure 1C). These results demonstrated that the lesion microenvironment triggered cytotoxic programs in effector CD8^+^ T cells.

To determine the mechanisms by which CD8^+^ T cell cytotoxic programs are induced in lesions, we performed RNA-Seq analysis on purified antigen-experienced CD8^+^ T cells from dLNs and lesions of mice infected with *Leishmania* for 5 weeks. Principal component analysis (PCA) showed that almost half (principal component 1 [PC1], 47%) of the differences on the whole transcriptional profiles were associated with the organ from which the CD8^+^ T cells were derived (Figure 1D). Differentially expressed gene (DEG) analysis with thresholds of a FDR of less than 0.05 and a fold change (FC) of greater than 1.5 between tissues revealed overexpression of 118 genes in CD8^+^ T cells from lesions compared with dLNs and 26 DEGs overexpressed in dLNs compared with lesions (Figure 1E, Supplemental Table 1, and Supplemental Figure 1, C and D). *Gzmb* was the top DEG in CD8^+^ T cells from lesions compared with dLNs, with an FDR of 0.002 (Figure 1E and Supplemental Figure 1C). Several transcription factors play important roles in effector CD8^+^ T cell biology, including BATF, ID-2, IRF4, Stat4, T-bet, Zeb2, and Blimp-1 (encoded by *Prdm1*) (58–60), and we found that *Id2*, *Irf4*, and *Prdm1* expression levels were significantly higher in lesions than in dLNs and that *Prdm1* had the most significant FC between tissues (>100 FC higher in lesions) (Figure 1F). To further investigate the signals received by CD8^+^ T cells within lesions, we performed gene set enrichment analysis (GSEA) (Supplemental Table 2), which revealed an array of immune-related pathways, including a hypoxia-related signature (Figure 1G). Hypoxia is a common feature of many inflammatory disorders and can alter CD8^+^ T cell function (38, 61). To determine whether hypoxia is a critical signature of lesional CD8^+^ T cells, we assessed other pathway databases, as well as the Harris et al. hypoxia-specific pathway described in ref. 62, to see if there was enrichment for a hypoxia signature in lesional compared with dLN CD8^+^ T cells. We found enrichment for multiple hypoxia-related signatures in CD8^+^ T cells in lesions (Figure 1H and Supplemental Table 2). These data indicate that, after their exit from dLNs, CD8^+^ T cells recruited to cutaneous leishmaniasis lesions were exposed to a hypoxic environment, inducing the expression of key cytotoxicity effector mediators, including *Prdm1*.

### Cutaneous leishmaniasis lesions are hypoxic and alter CD8^+^ T cell function.

**Figure 2:**
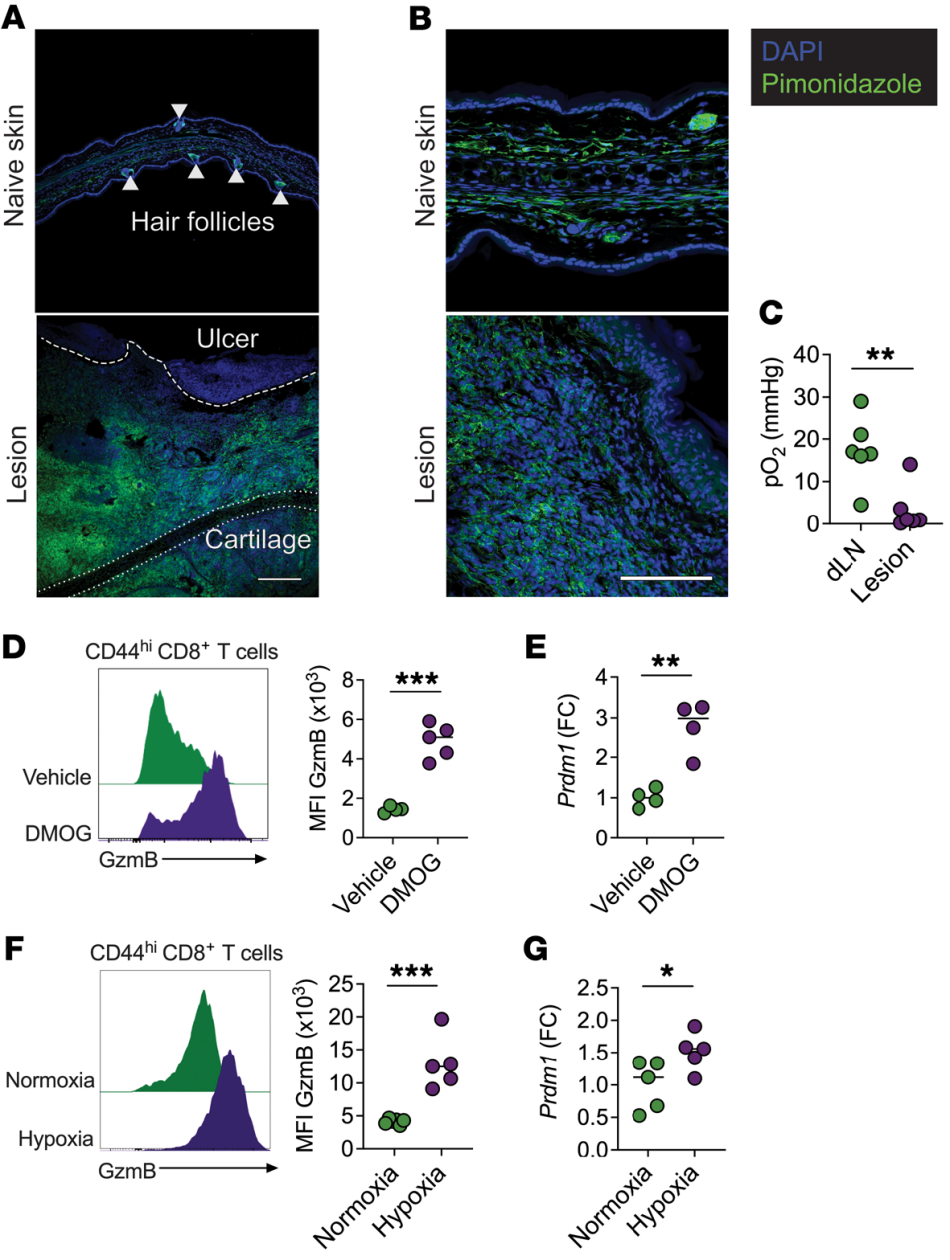
Hypoxia induces GzmB and *Prdm1* expression in CD8^+^ T cells. (**A** and **B**) Immunofluorescence staining and confocal microscopy of horizontal sections from lesions of C57BL/6 mice infected with *L. major* for 2 weeks. Mice received pimonidazole 1 hour before euthanasia. The top panels show contralateral ears (naive skin), and the bottom panels show lesions from infected ears. Pimonidazole is shown in green and nuclear DAPI staining in blue. Representative images are from 2 naive ears and 3 infected ears. Scale bars: 200 μm (**A**) and 100 μm (**B**). (**C**) Partial pressure of oxygen (pO_2_) in mmHg in dLNs and lesions of C57BL/6 mice infected with *L*. *major* for 2 weeks. Data were combined from 2 independent experiments. (**D**) GzmB expression in CD8^+^ T cells from dLNs of *L*. *major*–infected C57BL/6 mice; cells were cultured with DMOG or vehicle. (**E**) FC of *Prdm1* mRNA over the average expression of vehicle-treated cells measured by qRT-PCR in DMOG- or vehicle-treated purified CD8^+^ T cells. (**F**) GzmB expression in CD8^+^ T cells from dLNs of C57BL/6 mice infected with *L*. *major*; cells were cultured under normoxia (21% O_2_) or hypoxia (1% O_2_). (**G**) FC of *Prdm1* mRNA over the average expression of normoxic cells measured by qRT-PCR in purified CD8^+^ T cells cultured in normoxia or hypoxia. (**D**–**G**) Data shown are representative of more than 3 experiments with at least 4 mice per experiment. *P ≤ 0.05, **P ≤ 0.01, and ***P ≤ 0.001, by 2-tailed Student’s *t* test (**C**–**G**).

Normal skin is naturally low in O_2_ (63, 64), and since inflammatory environments are frequently hypoxic (65), we hypothesized that the lesions would be hypoxic. To evaluate hypoxia in *Leishmania*-infected lesions, we used pimonidazole, a 2-nitroimidazole reporter molecule that is reductively activated and forms covalent bonds with macromolecules in hypoxic cells. Two weeks after *Leishmania* infection, we assessed pimonidazole staining in infected and contralateral ears by confocal microscopy. As expected, we found pimonidazole staining within the epidermis, dermis, and hair follicles of naive mice (Figure 2, A and B, top images). Within lesions, we observed enlargement of the epidermis and dermis associated with robust pimonidazole staining (Figure 2, A and B, bottom images). Pimonidazole staining was absent from ulcerated areas, suggesting tissue heterogeneity in the hypoxic state of lesions. Representative images at lower (Figure 2A and Supplemental Figure 2A) and higher (Figure 2B and Supplemental Figure 2B) magnification show the differences in pimonidazole staining distribution between the naive and infected tissues.

To compare O_2_ levels in lesions and dLNs, we infected mice with *Leishmania,* and 2 weeks after infection, mice received Oxyphor G4, a phosphorescent probe quenched by O_2_ (66). Infected skin showed a significant decrease in O_2_ compared with dLNs (Figure 2C). These data directly demonstrated that skin lesions present a hypoxic microenvironment to CD8^+^ T cells upon their recruitment from dLNs.

We tested whether exposure to hypoxia is sufficient to promote GzmB expression in dLN CD8^+^ T cells. We isolated dLN cells from infected mice and stimulated them with anti-CD3 and anti-CD28 antibodies in the presence of dimethyloxalylglycine (DMOG), a compound that mimics hypoxia at normal O_2_ tension. As shown in Figure 2D, DMOG induced the expression of GzmB in CD8^+^ T cells, further demonstrating that mimicking hypoxia promotes upregulation of their cytolytic program. Notably, DMOG treatment also increased *Prdm1* mRNA expression compared with vehicle-treated CD8^+^ T cells (Figure 2E). We confirmed these findings by exposing CD8^+^ T cells to 1% O_2_ and found that hypoxia induced GzmB and *Prdm1* expression in CD8^+^ T cells (Figure 2, F and G). We also tested whether hypoxia had a similar effect on naive and antigen-experienced CD8^+^ T cells (Supplemental Figure 3A) and we found that hypoxia enhanced GzmB expression only in antigen-experienced CD8^+^ T cells (Supplemental Figure 3B). These results raise the question of whether CD8^+^ T cells in vivo require *Leishmania* antigen to express GzmB or if sustained hypoxia alone is sufficient to promote GzmB. To test this, we infected mice with a T cell conditional deletion of Von Hippel–Lindau (referred to here as VHL^cKO^ mice), which is necessary for the degradation of hypoxia-inducible factors (HIFs). Consequently, VHL^cKO^ mice have HIF stabilization even in normoxic conditions. VHL deficiency did not increase GzmB expression in naive skin (Supplemental Figure 3C), suggesting that without *Leishmania*, there was no induction of GzmB when cells are forced to respond to hypoxia. In infected ears, VHL deletion increased GzmB expression in CD8^+^ T cells (Supplemental Figure 3C). One possible explanation is that at least a portion of CD8^+^ T cells in lesions were not exposed to hypoxia, which agrees with our observation that there were varied degrees of pimonidazole staining in the infected skin (Figure 2A). Another possibility is that CD8^+^ T cells newly recruited from the blood were not fully hypoxic and that forcing an immediate hypoxic response induced GzmB in these cells. Nevertheless, these data showed that antigen stimulation was necessary for CD8^+^ T cells to express GzmB in hypoxic conditions.

While the primary role of CD4^+^ T cells in our model is the production of IFN-γ and protection (1), CD4^+^ T cells can also be cytotoxic (67). Therefore, we tested whether hypoxia (1% O_2_) induces GzmB expression in CD4^+^ T cells from the dLNs of infected mice stimulated with anti-CD3 and anti-CD28 antibodies. Naive and antigen-experienced CD4^+^ T cells expressed more GzmB when exposed to hypoxia (Supplemental Figure 3D). However, GzmB expression was lower in CD4^+^ T cells than in CD8^+^ T cells (Supplemental Figure 3, B and D), suggesting that the induction of GzmB by hypoxia was shared by CD4^+^ and CD8^+^ T cells; however, the magnitude of the response was greater in CD8^+^ T cells. Given that CD4^+^ T cells were also exposed to the hypoxic microenvironment of lesions, we analyzed GzmB expression in CD4^+^ T cells in dLNs and lesions. We found that CD4^+^ and CD8^+^ T cells had a similar profile, with higher GzmB expression in lesions, although CD4^+^ T cells expressed significantly less GzmB than did CD8^+^ T cells (Supplemental Figure 3E). Collectively, these data demonstrate that the hypoxic state of lesions combined with antigen exposure stimulated the differentiation of recruited CD8^+^ T cells into cytotoxic effectors, with a moderate effect on the development of cytotoxic CD4^+^ T cells.

### Blimp1 expression is restricted to GzmB-expressing CD8^+^ T cells.

**Figure 3:**
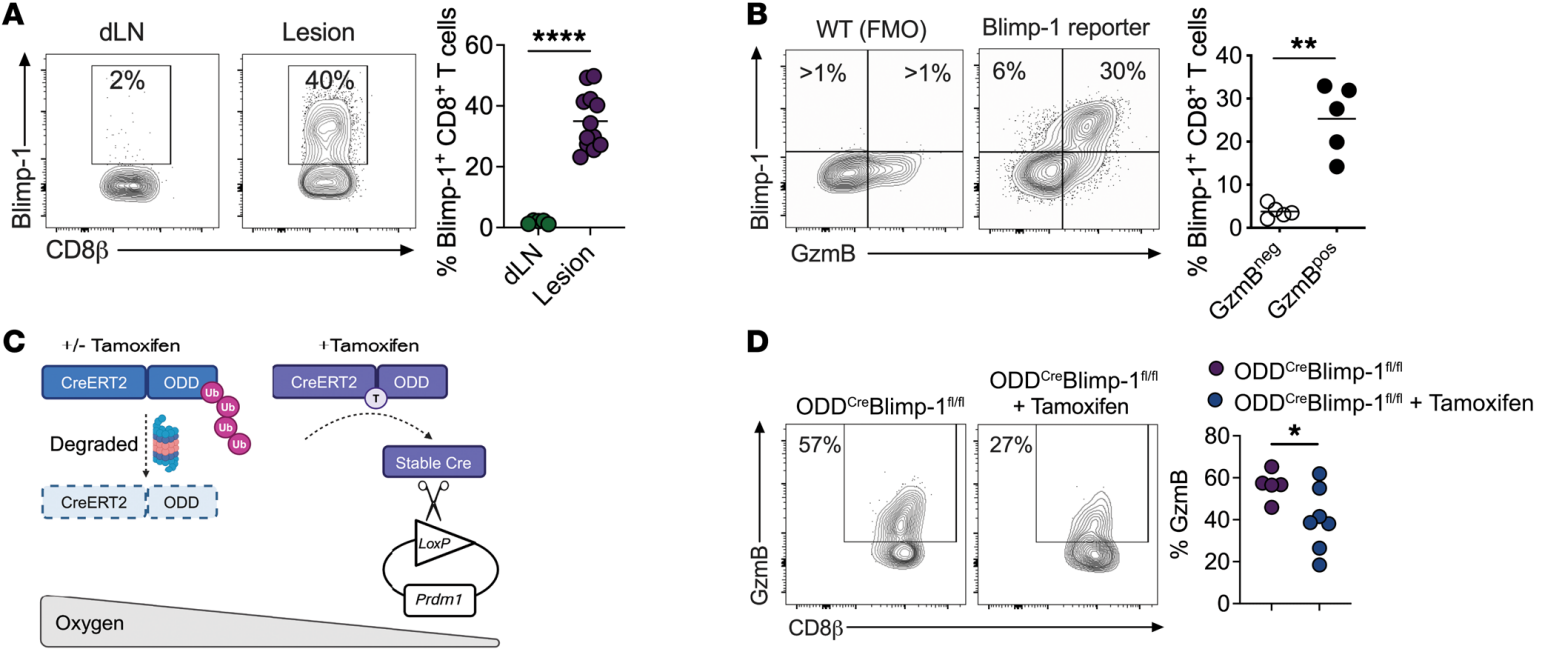
Blimp-1 expression is induced by hypoxia within skin lesions. (**A**) Expression of Blimp-1 in CD8^+^ T cells from dLNs and lesions from Blimp-1 YFP reporter mice infected with *L*. *major* for 1 week. Data from 2 independent experiments combined. (**B**) GzmB and Blimp-1 YFP expression in CD8^+^ T cells from lesions from WT or Blimp-1 reporter mice infected with *L*. *major* for 1 week. (**C**) Schematic representation of ODD^Cre^ Blimp-1^fl/fl^ mice. (**D**) ODD^Cre^ Blimp-1^fl/fl^ mice had been infected with *L*. *major* for 1 week when daily tamoxifen injections commenced. Mice were euthanized 2 weeks after infection. GzmB expression of lesional CD8^+^ T cells in tamoxifen-treated and untreated mice. Data were combined from 2 independent experiments. *P ≤ 0.05, **P ≤ 0.01, and ****P ≤ 0.0001, by 2-tailed Student’s *t* test (**A**, **B,** and **D**).

Blimp-1–deficient CD8^+^ T cells produce less GzmB (68), so we next sought to test cause-effect relationships between hypoxia, Blimp-1, and cytotoxicity. To this end, we infected Blimp-1 yellow fluorescent protein (YFP) reporter mice with *Leishmania* and assessed Blimp-1 expression in CD8^+^ T cells. We found that Blimp-1 was highly expressed in lesional CD8^+^ T cells but not in CD8^+^ T cells from the dLNs (Figure 3A). We also found that Blimp-1 expression was significantly higher in CD8^+^ T cells that expressed GzmB (Figure 3B), with similar results in CD4^+^ T cells (Supplemental Figure 4, A and B), suggesting a link between Blimp-1 and cytotoxicity in both T cell subsets. Since Blimp-1 regulates IL-10 production in CD4^+^ T cells (69), we measured the expression levels of IL-10 in dLNs and lesions of infected mice and found higher expression of IL-10 in lesional CD4^+^ and CD8^+^ T cells compared with dLNs (Supplemental Figure 4, C and D). In lesions, CD4^+^ and CD8^+^ T cells had higher Blimp-1 expression in IL-10^pos^ cells than did their IL-10^neg^ counterparts (Supplemental Figure 4, E and F), suggesting a strong relationship between Blimp-1 expression and IL-10, as previously described (69).

Finally, to assess whether GzmB expression in lesional CD8^+^ T cells resulted from hypoxic induction of Blimp-1, we used mice expressing a fusion protein bearing an oxygen-dependent degradation (ODD) domain from human HIF1A fused to a tamoxifen-inducible Cre recombinase gene (Cre/ERT2) (70). When expressed under normoxic conditions, the O_2_CreER fusion protein was degraded rapidly but was stabilized by hypoxia when combined with tamoxifen injection. These mice were crossed with Blimp-1^fl/fl^ mice (referred to here as ODD^Cre^ Blimp-1^fl/fl^ mice) to generate mice in which Blimp-1 expression is deleted specifically in hypoxic cells upon tamoxifen injection (Figure 3C). Since the skin has significantly less O_2_ than dLNs, these mice provide lesion-specific deletion of Blimp-1, thus ensuring the preservation of Blimp-1–dependent development of effector T cells in dLNs (normoxic) and their recruitment to the inflamed tissue. *Leishmania*-infected ODD^Cre^ Blimp-1^fl/fl^ mice were treated with tamoxifen or left untreated for 1 week, and GzmB expression was evaluated. We found that the specific deletion of Blimp-1 in hypoxic cells significantly decreased GzmB expression in lesional CD8^+^ T cells compared with untreated mice (Figure 3D). Together, these results demonstrate that the upregulation of Blimp-1 by the hypoxic environment led to increased GzmB expression in CD8^+^ T cells.

### A subset of GzmB^pos^ CD8^+^ T cells express markers of exhaustion.

Both hypoxia and Blimp-1 can induce the expression of many coinhibitory receptors involved in the exhaustion program (68, 71–74). To verify whether CD8^+^ T cells in lesions are exhausted, we reanalyzed a publicly available bulk RNA-Seq data set of 3 subsets of activated (stem-like CD101^neg^Tim3^neg^, transitory CD101^neg^Tim3^pos^ and exhausted CD101^pos^Tim3^pos^) PD-1^pos^ CD8^+^ T cells and naive CD8^+^ T cells in the context of chronic lymphocytic choriomeningitis virus (LCMV) infection. On the basis these subsets, we generated 3 specific gene signatures and compared them with our data set of CD8^+^ T cells isolated from the dLN and lesions (Figure 1, D–H). CD8^+^ T cells isolated from the lesions were enriched for all 3 signatures compared with CD8^+^ T cells isolated from the dLN (Supplemental Figure 5A). A subset of genes associated with exhaustion was higher in cells from lesions compared with dLNs, such as *Pdcd1* (encoding for PD-1), *Lag3* (encoding for Lag3), and *Havcr3* (encoding for Tim3), while other genes — *Tox*, *Cx3cr1*, or *Tcf7* — were not differentially represented in either tissue (Supplemental Figure 5B). We confirmed increased protein expression of PD-1, Lag3, and Tim3 in CD8^+^ T cells from lesions 2 weeks after infection (Supplemental Figure 5C). We sought to determine whether there was preferential expression of PD-1, Lag3, or Tim3 in CD8^+^ T cells that were GzmB^pos^ and, therefore, had higher Blimp-1 expression and found that only Tim3 was preferentially expressed in GzmB-expressing CD8^+^ T cells (Supplemental Figure 5D). Collectively, these data suggest that coinhibitory molecule expression was a feature of some CD8^+^ T cells present in lesions and that a small subset of GzmB^pos^ CD8^+^ T cells were either terminally exhausted or on their way to becoming so.

### Blimp-1 expression is necessary for CD8^+^ T cells to mediate disease.

**Figure 4:**
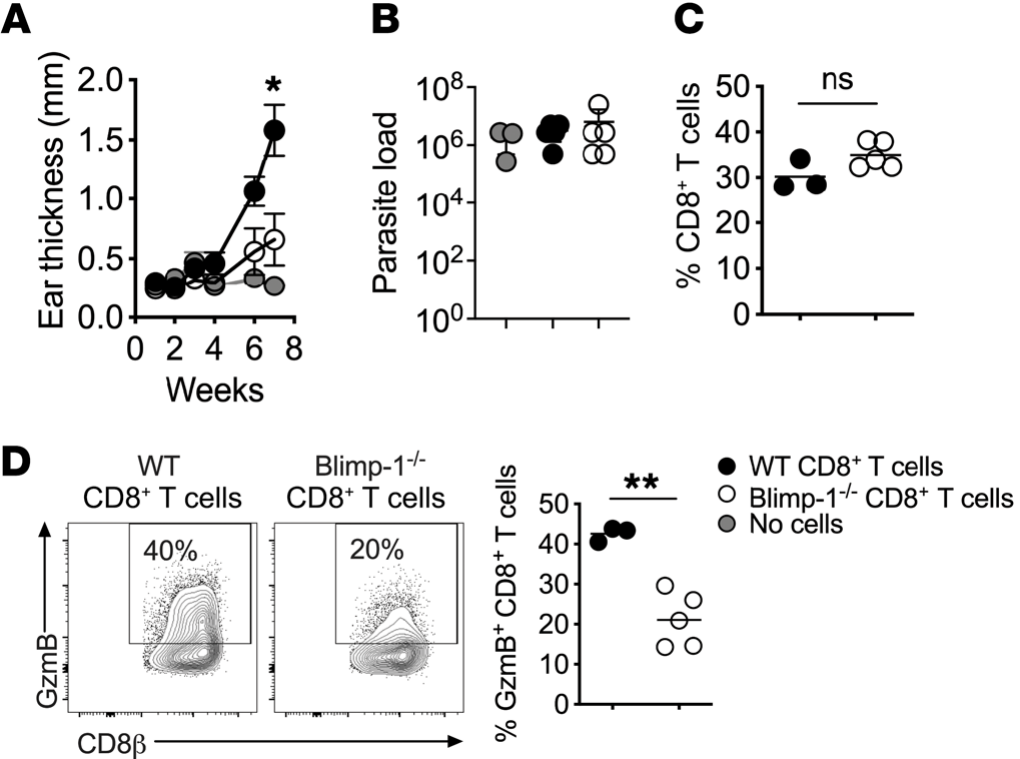
Blimp-1 expression is required for CD8^+^ T cell–mediated disease. RAG^−/−^ mice were infected with *L*. *braziliensis* and reconstituted with purified CD8^+^ T cells from WT or Blimp-1^cKO^ mice. (**A**) Ear thickness and (**B**) parasite numbers in lesions at 7 weeks of infection. (**C**) CD8^+^ T cell frequency and (**D**) GzmB expression by CD8^+^ T cells in lesions were assessed directly ex vivo by flow cytometry 7 weeks after infection. Data represent 3 individual experiments with 3–5 mice per group. *P ≤ 0.05 and **P ≤ 0.01, by 2-tailed Student’s *t* test (**A**, **C**, and **D**).

On the basis of our data, we predicted that Blimp-1 expression is required for cytotoxic CD8 T cell–mediated disease in leishmaniasis. Blimp-1 plays a vital role in CD4^+^ T cell subsets (69, 75); hence, total T cell deletion of Blimp-1 would complicate interpretations. Therefore, we used our well-characterized mouse model of chronic leishmaniasis, in which severe disease develops following *Leishmania* infection of RAG^–/–^ mice reconstituted with CD8^+^ T cells (3, 4, 55, 76). In this model, pathology is dependent on the ability of CD8^+^ T cells to be cytotoxic (3). RAG^–/–^ mice infected with *Leishmania* were reconstituted with WT or CD8^+^ T cells lacking Blimp-1 expression (referred to here as Blimp-1^cKO^ mice) or control animals receiving no T cells. As described previously (3), RAG^–/–^ mice reconstituted with WT CD8^+^ T cells developed severe pathology characterized by GzmB-expressing CD8^+^ T cells in lesions, whereas RAG^–/–^ mice that received no cells showed no signs of pathology (Figure 4A). Importantly, RAG^–/–^ mice reconstituted with Blimp-1^cKO^ CD8^+^ T cells had minimal disease (Figure 4A). As expected, there were similar numbers of parasites (Figure 4B) and a similar frequency of CD8^+^ T cells (Figure 4C) in lesions of RAG^–/–^ mice reconstituted with WT and Blimp-1^cKO^ CD8^+^ T cells. Notably, GzmB expression was significantly diminished in cells lacking Blimp-1 (Figure 4D). Collectively, these data indicate that Blimp-1 expression was driven by the hypoxic microenvironment of the lesion, triggering GzmB expression and CD8^+^ T cell–driven pathology.

### Neutrophils generate the hypoxic microenvironment of cutaneous leishmaniasis lesions.

**Figure 5:**
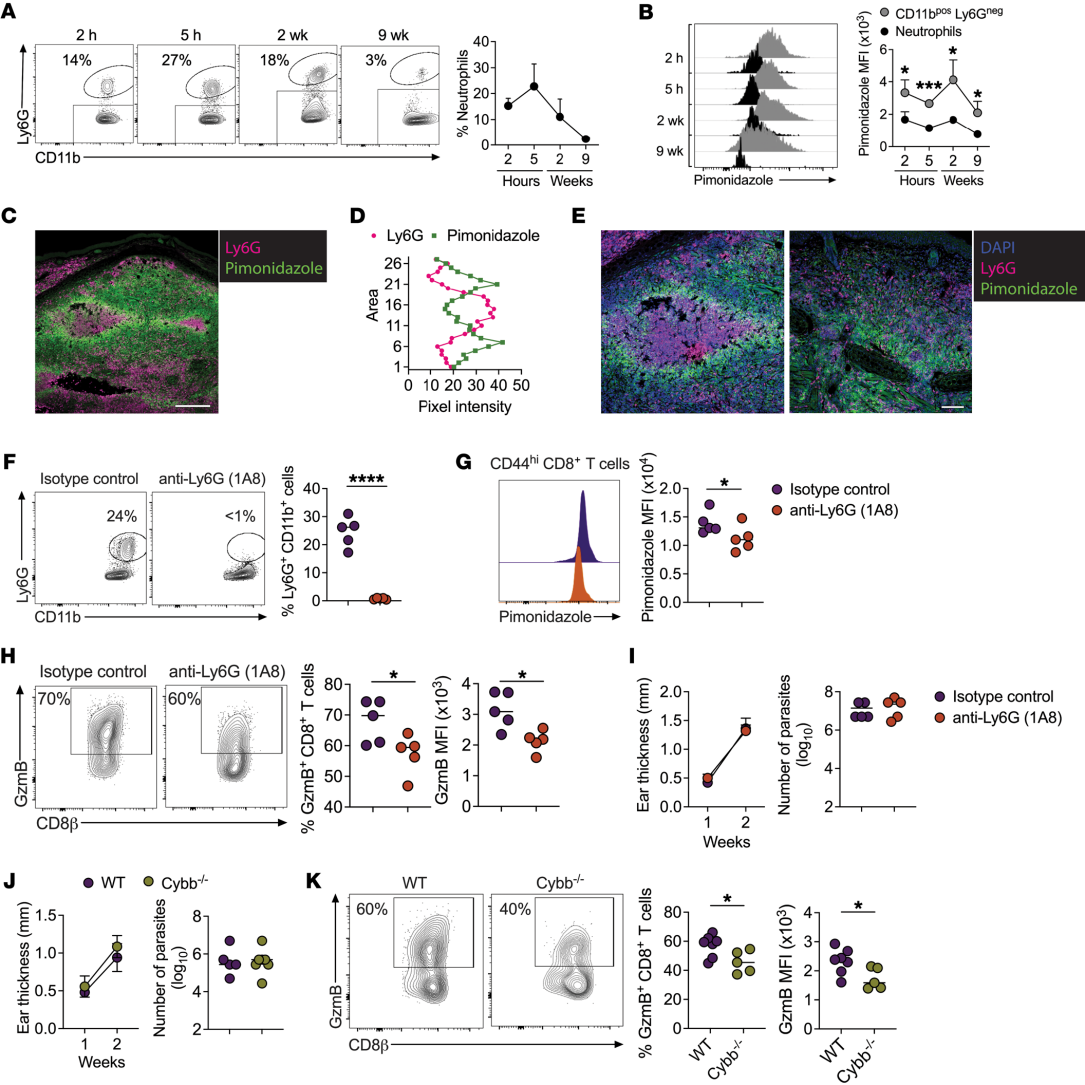
Neutrophils consume oxygen from lesions and stimulate GzmB expression in CD8^+^ T cells. (**A** and **B**) C57BL/6 mice infected with *L*. *major* received pimonidazole 1 hour before euthanasia at 2 hours, 5 hours, 2 weeks, and 9 weeks. (**A**) Frequency of neutrophils in lesions and (**B**) pimonidazole expression in neutrophils (CD11b^+^Ly6G^pos^) and other myeloid cells (CD11b^+^Ly6G^neg^) in lesions. (**C**) Representative confocal microscopy image of Ly6G (pink) and pimonidazole (green) staining in skin infected for 2 weeks. Scale bar: 200 μm. (**D**) Pixel intensity of pimonidazole and Ly6G based on 27 regions (Supplemental Figure 6B). (**E**) Two representative confocal microscopy images of nuclear staining (DAPI, blue), Ly6G (pink), and pimonidazole (green) in lesions infected for 2 weeks. Scale bar: 100 μm. (**F**–**I**) C57BL/6 mice infected with *L*. *major* were injected every 3 days with anti-Ly6G (clone 1A8) antibody or isotype control until euthanasia 2 weeks after infection. (**F**) Frequency of neutrophils in lesions, (**G**) pimonidazole staining in CD8^+^ T cells in lesions, and (**H**) GzmB frequency and MFI in CD8^+^ T cells in lesions. (**I**) Lesion size and number of parasites in lesions. (**J**) Lesion size and number of parasites in lesions in WT and Cybb^–/–^ mice infected with *L*. *major* for 2 weeks. (**K**) GzmB frequency and MFI in CD8^+^ T cells in the lesions of WT or Cybb^–/–^ mice. *P ≤ 0.05, ***P ≤ 0.001, and ****P ≤ 0.0001, by 2-tailed Student’s *t* test (**B**, **F**, **H**, and **K**) and 1-tailed Student’s *t* test (**G**).

Hypoxia occurs in tissues when O_2_ supply does not meet demand, including scenarios characterized by defective tissue vascularization or increased demand by cells present within a tissue (38). *Leishmania*-infected lesions are highly vascularized (77), so we hypothesized that O_2_ consumption by inflammatory cells recruited to lesions promotes hypoxia. Neutrophils are the first cells recruited to the skin upon *Leishmania* infection (78), but their recruitment does not control *L*. *major* parasites. Indeed, their chronic presence is associated with worsening disease (3, 4, 79–83). Therefore, we tested whether neutrophil recruitment contributed to the hypoxic state of inflamed skin. *Leishmania*-infected mice were injected with pimonidazole 1 hour before euthanasia at multiple time points after infection: (a) soon after parasite challenge (2 and 5 hours), (b) when lesions started to develop and CD8^+^ T cells were present and expressed GzmB (2 weeks), and (c) when lesions began to heal (9 weeks). As expected (78), neutrophils were quickly recruited after infection and decreased in frequency when lesions resolved (Figure 5A). Surprisingly, neutrophil (CD11b^pos^Ly6G^pos^) staining by pimonidazole was significantly lower compared with other myeloid cells (CD11b^pos^Ly6G^neg^) in the skin at all time points analyzed (Figure 5B). To confirm these observations, lesions from pimonidazole-injected mice were stained for Ly6G, revealing intense neutrophil recruitment in ulcered regions in the epidermis and clusters of neutrophils within the skin dermis (Figure 5C and Supplemental Figure 6A). Notably, regions without neutrophils had more pimonidazole staining than did those containing neutrophil clusters. We also observed that pimonidazole staining in regions surrounding neutrophil clusters was more intense. A quantitative assessment of pixel intensities (Supplemental Figure 6B) revealed a negative correlation between pimonidazole and Ly6G expression (Figure 5D). Higher magnification of regions with intense neutrophil recruitment in 2 samples showed a lack of pimonidazole staining in neutrophils (Figure 5E and Supplemental Figure 6C).

These results suggest that areas surrounding neutrophils were relatively hypoxic. Accordingly, we hypothesized that neutrophils are responsible for promoting the hypoxic state of the skin by competing for oxygen, thereby controlling CD8^+^ T cell effector function. To test this, we depleted mice of neutrophils for the first 2 weeks of infection using anti-Ly6G antibody (Figure 5F) and assessed pimonidazole and GzmB expression in CD8^+^ T cells. We found that neutrophil depletion decreased pimonidazole expression (Figure 5G) and the frequency and MFI of GzmB-expressing CD8^+^ T cells compared with isotype control–treated mice (Figure 5H), without changing lesion size or parasite numbers (Figure 5I). These data suggest that neutrophils consumed O_2_ in lesions, altering the phenotype of CD8^+^ T cells.

Activated neutrophils assemble NADPH oxidase to produce ROS, which requires O_2_ (84). Thus, we tested whether O_2_ consumption to generate ROS affected CD8^+^ T cell function. For this purpose, we compared the ability of lesional CD8^+^ T cells to express GzmB in mice sufficient (WT) or deficient in the gp91^phox^ subunit of the NADPH oxidase (Cybb^–/–^). Although there was no change in lesion size or parasite numbers in the skin at week 2 after infection (Figure 5J), we found significantly lower frequencies of GzmB-expressing CD8^+^ T cells in Cybb^–/–^ mice than in WT (Figure 5K), forging a link between NADPH oxidase–dependent ROS production and the development of pathogenic CD8^+^ T cells in cutaneous leishmaniasis lesions.

### The magnitude of hypoxia correlates with the presence of neutrophils in human cutaneous leishmaniasis lesions.

**Figure 6:**
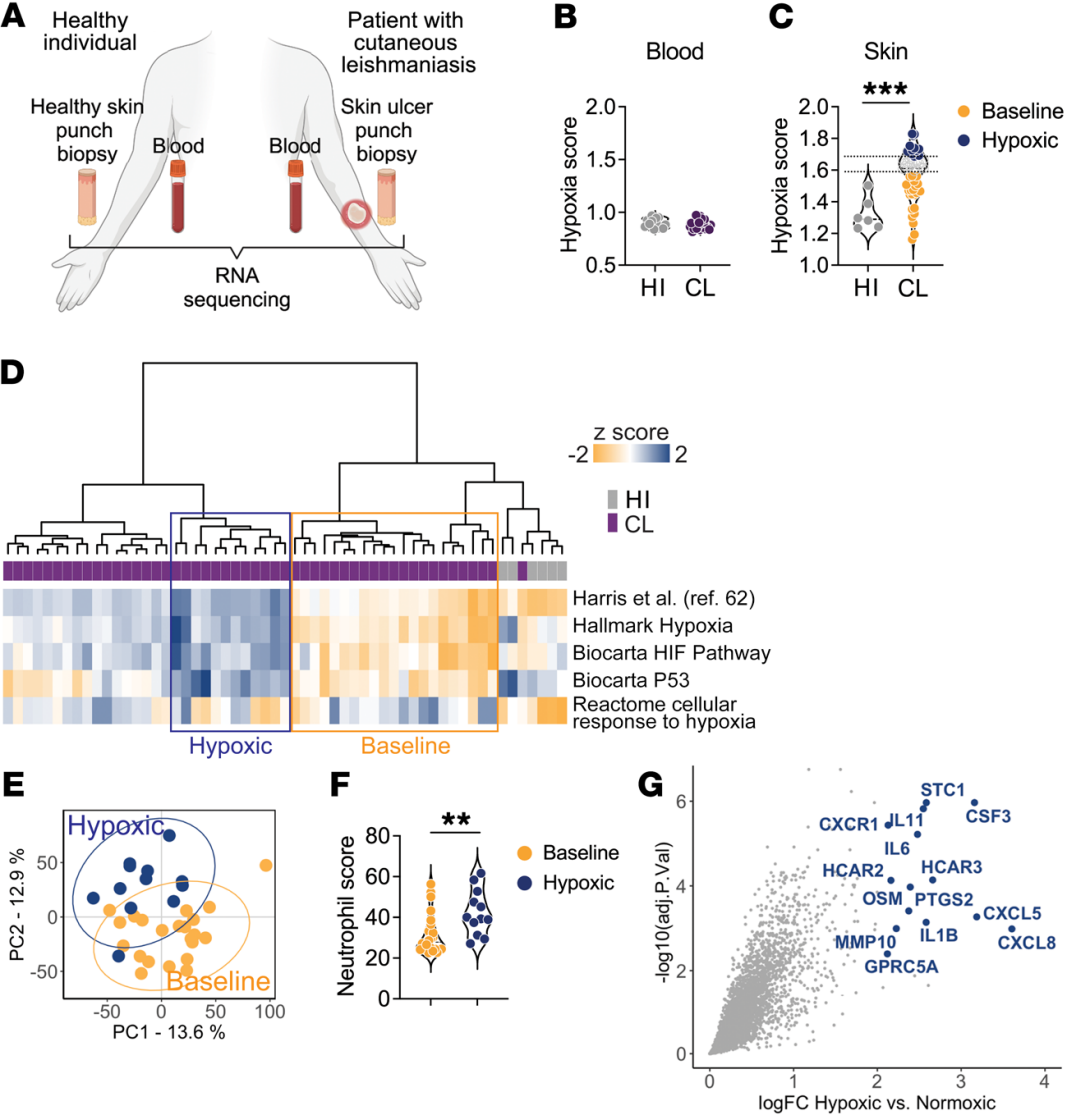
The degree of hypoxia-related gene expression is associated with the presence of neutrophils in patients. (**A**) Schematics of data collection for the RNA-Seq analysis performed in whole blood and total skin punch biopsies in healthy individuals and patients with cutaneous leishmaniasis. (**B**) Hypoxia score calculated in blood from healthy individuals (HI) (*n* = 14) and patients with cutaneous leishmaniasis (CL) (*n* = 50) and in (**C**) human intact skin (*n* = 6) and cutaneous leishmaniasis lesions from patients (*n* = 51) based on the Harris et al. (62) hypoxia gene signature. (**D**) Unsupervised hierarchical clustering classification of human skin samples in “baseline” and “hypoxic” lesions according to their hypoxia gene phenotypes. (**E**) PCA of baseline and hypoxic cutaneous leishmaniasis lesions. (**F**) Neutrophil abundances estimated by the MCP-counter method. The Wilcoxon test was used for statistical analysis. (**G**) Scatter plot showing differential gene expression analysis between hypoxic versus baseline lesions (FDR > 0.01 and FC = 1.5). In blue are the top DGEs from this analysis. **P ≤ 0.01 and ***P ≤ 0.001, by 2-tailed Student’s *t* test (**C** and **F**). HI, healthy individuals.

To determine whether hypoxia is a feature of human disease, we compared transcriptional signatures from bulk RNA-Seq analysis performed in whole blood and total skin from healthy individuals and patients with cutaneous leishmaniasis using previously published data sets (54, 85) (Figure 6A). To estimate the levels of hypoxia gene expression in clinical samples, we performed single-sample GSEA using the Harris et al. hypoxia gene signature (62) and referred to this variable as the “hypoxia score” (Supplemental Table 3). While there was no difference in the hypoxia scores between blood from patients with cutaneous leishmaniasis and healthy individuals (Figure 6B and Supplemental Table 4), the cutaneous leishmaniasis lesions were significantly more hypoxic than healthy skin (Figure 6C and Supplemental Table 3). Importantly, we observed that the degree of the hypoxia-related gene expression in patients’ lesions was variable. To understand the biological implication of this variability, we performed unsupervised hierarchical clustering (HC) to classify lesions and healthy skin samples according to their hypoxic gene expression (Figure 6D). HC analysis revealed 2 groups of lesion samples: (a) “baseline” (*n* = 22), with a hypoxia phenotype comparable to that of control skin samples, and (b) “hypoxic” (*n* = 12), with a high hypoxic phenotype relative to the entire cohort (Figure 6, C and D). The remaining lesions had an intermediate phenotype. PCA revealed that hypoxic lesions were robustly segregated from the baseline counterparts: permutational multivariate ANOVA (PERMANOVA) (Pr), Pr(> *F*) = 0.001) (Figure 6E). Gene Ontology performed for genes overexpressed in hypoxic versus baseline lesions (FDR > 0.01 and FC = 1.5 thresholds) revealed significant enrichment for neutrophil chemotaxis and signaling (Supplemental Tables 5 and 6). Indeed, estimation of cell abundances from this unstructured bulk RNA-Seq data revealed an increase in neutrophil counts in hypoxic versus baseline lesions (Figure 6F). The top DEGs between hypoxic compared with baseline lesions included genes associated with neutrophil chemotaxis and survival, such as *CXCL5*, *CXCL8*, *CXCR1*, *CSF3*, and genes encoding proinflammatory cytokines such as *IL1B*, *IL6*, and *OSM* (Figure 6G and Supplemental Table 5). Therefore, our findings in mice are supported by our analysis of patients’ samples, linking the presence of neutrophils to the degree of hypoxia in lesions.

## Discussion

Cytolytic CD8^+^ T cells cause severe inflammation in various settings (14, 15, 21, 24, 31–34, 86), although the factors that promote cytolysis by CD8^+^ T cells in the tissue microenvironments are still poorly understood. Here, we show that neutrophils recruited to the skin upon *Leishmania* infection consumed O_2_ to produce ROS, generating chronic inflammatory hypoxia. The resultant competition for O_2_ changed the aerobic microenvironment and promoted the expression of Blimp-1 in recruited CD8^+^ T cells, which resulted in GzmB expression and tissue damage. Because numerous skin diseases are associated with cytotoxicity, our findings provide evidence that local inhibition of this pathway should be considered as a strategy to ameliorate these diseases.

Preferential expression of cytolytic genes by CD8^+^ T cells in peripheral tissues but not in dLNs is observed in numerous conditions. For example, in models of graft-versus-host disease and graft-versus-leukemia, GzmB is absent in dLNs but present in the skin (9, 11). Additionally, memory CD8^+^ T cells acquire cytolytic activity upon entry into nonlymphoid tissues in vesicular stomatitis viral infections (10). The site-specific function of CD8^+^ T cells is not limited to GzmB expression. For example, in mice chronically infected with *Trypanosoma cruzi*, CD8^+^ T cells in the spleen produced IFN-γ, whereas CD8^+^ T cells within the muscle did not (87). Similarly, as previously shown (55), we found that CD8^+^ T cells in dLNs produced IFN-γ, while CD8^+^ T cells in the skin produced very little IFN-γ. Since hypoxic DCs produce less IL-12 (46, 48), this could explain the decrease in CD8^+^ T cell IFN-γ production in lesions (55) and suggests that hypoxia may not only promote pathogenic responses but may also prevent protective ones. Conversely, in diseases in which cytotoxicity is protective and IFN-γ is pathogenic, hypoxia would be expected to have the opposite effect. Our confocal microscopy data suggest that hypoxia affected many other cells beyond CD8^+^ T cells. In support of this observation, we found that CD4^+^ T cells also expressed more GzmB when exposed to hypoxia; however, the relevance of these cytotoxic CD4^+^ T cells to cutaneous leishmaniasis disease is unknown. Therefore, it is likely that hypoxia has a much greater role in tissue immunity to *Leishmania*.

There is substantial clinical and experimental evidence that hypoxia contributes to tumor progression, metastasis, and high mortality (88). While low O_2_ levels indicate myeloid suppressor cell accumulation (89) and tumor cell metastasis (90), HIFs provide CD8^+^ T cells with greater antitumor potential (91–93). HIF increases CD8^+^ T cell effector function during chronic viral infections and reduces viral titers, leading to higher mortality (94). These findings agree with the observed increases in perforin expression and killing ability of CD8^+^ T cells cultured in hypoxic conditions (93, 95, 96). Similar results were observed in lupus skin disease, in which HIF drove GzmB expression but, in this case, was independent of hypoxia (97). Hypoxia also alters CD8^+^ T cell metabolism (95) and induces tissue residency (91, 98) and exhaustion (72–74) programs. One of the limitations of our work is that it does not address the effect of HIFs on GzmB expression, and it is possible that HIFs directly promote *Gzmb*. However, our data provide evidence of an indirect induction of GzmB by hypoxia through Blimp-1 expression. Consistent with our findings, hypoxia also triggered Blimp-1 expression in pancreatic tumor cells (90). Since Blimp-1 can instruct tissue residency and exhaustion markers (68, 99–101), and we found an increased exhaustion phenotype in CD8^+^ T cells in the skin, we speculate that hypoxia-driven Blimp-1 expression may also initiate the CD8^+^ T cell program of exhaustion within cutaneous leishmaniasis lesions, which requires further investigation.

The effect of hypoxia is contradictory, as it produces more cytotoxic CD8^+^ T cells, while suppressing the function of some myeloid cells (46, 48). This balance may also occur within the same cell: for example, hypoxic induction of Blimp-1 regulates the cytotoxic function in T cells and paradoxically increases the expression of the antiinflammatory cytokine IL-10. In some situations, Blimp-1–dependent IL-10 production by CD4^+^ T cells protects against tissue damage (69). In CD8^+^ T cells, the cytotoxic program induced by Blimp-1 appears to outweigh the antiinflammatory effect of IL-10 on cutaneous leishmaniasis.

Another immune cell type implicated in the hypoxia/cytotoxicity axis revealed in our study is neutrophils, which rapidly migrate to infection sites and, in many infections, eliminate invading microbes. However, it is well recognized that the effect of neutrophils extends beyond pathogen killing, as they also shape the function of other cells, consequently affecting disease outcomes. For example, neutrophils producing IL-10 (102) or expressing PD-L1 (103) suppress CD4^+^ T cells. Additionally, neutrophils suppress effector responses by tumor-specific T cells in LNs (104) and within tumors (105). In chronic viral infections, suppressive neutrophil subsets reduce the antiviral capabilities of CD8^+^ T cells (106). In contrast, neutrophils facilitate DC migration to dLNs in contact hypersensitivity and prime allergen-specific CD8^+^ T cells (107). In cutaneous leishmaniasis, neutrophils recruited to lesions can have conflicting functions depending on the parasite species and mouse model. For example, neutrophils kill *Leishmania* directly by extracellular trap formation or cooperate with macrophages to activate microbicidal activities (108, 109). In contrast, neutrophils reduce costimulatory molecule expression in DCs and silently deliver parasites to macrophages (78, 110, 111). Despite these varied effects, it is clear that chronic recruitment of neutrophils is associated with tissue destruction in cutaneous leishmaniasis (3, 81, 83, 112). Here, we uncovered a new role for neutrophils in cutaneous leishmaniasis lesions by showing that their O_2_ consumption caused a phenotypic switch in CD8^+^ T cells.

Cytotoxic CD8^+^ T cells promote increased inflammation by inducing excessive cell death in lesions and enhancing chronic neutrophil recruitment to the skin (3, 4). In what may be related findings, neutrophil depletion decreases the hypoxic state of herpes stromal keratitis lesions and ameliorates disease (113). In a mouse model of colitis, the respiratory burst by Gr1^pos^ cells — encompassing neutrophils and inflammatory monocytes — induced hypoxia in intestinal epithelial cells, which was critical for disease resolution (114). One limitation of our work is that it does not address the effect of other innate cells and their ability to deplete lesions of oxygen, which could also affect CD8^+^ T cell function. However, our work strongly supports the idea that O_2_ consumption and neutrophil recruitment are modulators of CD8^+^ T cell GzmB expression, which fosters the chronic presence of neutrophils in lesions (4). While the negative effect of this cascade of events is clear in cutaneous leishmaniasis, it could be beneficial in other contexts.

To be successful, host-directed therapies for infectious diseases should dampen immune responses that damage tissues and must not interfere with pathogen control (2). Therefore, defining immune pathways that are pathogenic rather than protective is critical. In cutaneous leishmaniasis, cytotoxicity is an ideal therapeutic target, since it is associated with treatment failure in patients who receive antiparasitic drugs (7) and does not control *Leishmania* (3). Patients affected by cutaneous leishmaniasis are frequently treated with antiparasitic drugs that require daily injections, and because failure rates are high, many patients must undergo multiple rounds of treatment. Our data provide evidence that local inhibition of the cytolytic program can circumvent the use of systemic drugs, which cause considerable side effects, and that topical targets should be considered as a new therapeutic strategy for cutaneous leishmaniasis.

## Methods

### Sex as a biological variable.

Our animal experiments were performed using male and female mice, and controls were sex matched, but sex was not specifically tested as a biological variable. Human donors of both sexes were used for RNA-Seq analysis, and no sex differences were detected.

### Mice.

C57BL/6 CD45.2 and CD45.1 mice were purchased from Charles River Laboratories, and *Prdm1* YFP, ODD^Cre^/ERT2 (70), dLck^Cre^, Blimp-1^fl/fl^, VHL^fl/fl^, and RAG^–/–^ (B6.12957-RAG1^tm1Mom^) mice were purchased from The Jackson Laboratory and crossed in our mouse facility. Cybb^–/–^ mice (115) were obtained from Juhi Bagaitkar (Nationwide Children’s Hospital, Columbus, Ohio, USA). Experiments assessing Blimp-1 expression with the Blimp-1 YFP reporter were reproduced using Blimp-1 GFP reporter mice (a gift from Stephen Nutt, Walter and Eliza Hall Institute of Medical Research [WEHI], Melbourne, Australia). Gzmb^Cre^ mice (obtained from John Wherry, University of Pennsylvania) have the human *GZMB* promoter directing Cre recombinase expression to activated T cells; therefore, it does not directly affect mouse GzmB expression. Gzmb^Cre^ and Blimp-1^fl/fl^ mice were crossed to generate the deletion of Blimp-1 in activated T cells, and here we called RAG^-/-^ mice that recieved purified CD8^+^ T cells from Gzmb^Cre^Blimp-1^fl/fl^ mice Blimp-1^cKO^. ODD^Cre^/ERT2 and Blimp-1^fl/fl^ mice were crossed to generate mice in which Blimp-1 deletion was restricted to hypoxic cells in tamoxifen-treated mice. dLck^Cre^ and VHL^fl/fl^ mice were crossed to generate the deletion of VHL in T cells after positive selection in the thymus, and we refer to these mice as VHL^cKO^ mice. dLck^Cre^ mice were used as WT controls. All mice were maintained in a specific pathogen–free environment at The Ohio State University or the University of Pennsylvania Animal Care Facilities.

### Parasites.

*L*. *major* (strain WHO/MHOM/IL/80/Friedlin) and *L*. *braziliensis* (MHOM/BR/01/BA788) were grown in Schneider’s insect medium (Gibco, Thermo Fisher Scientific) supplemented with 20% heat-inactivated FBS (MilliporeSigma) and 2 mM glutamine (Thermo Fisher Scientific). Metacyclic-enriched promastigotes were used for infection (116). Mice were infected with either 10^6^ *L*. *major* or 10^5^ *L*. *braziliensis* intradermally in the ear, and lesion progression was monitored weekly by measuring the diameter of the ear with a caliper.

### Cell purification and adoptive transfer.

For the intralesional cell transfer mouse model, CD45.2 and CD45.1 mice were infected with *L*. *major*. CD45.2 mice were euthanized 3 weeks after infection, and CD8^+^ T cells from dLNs were purified using a magnetic bead separation kit (Miltenyi Biotec). CD8^+^ T cells (10^6^ cells) were injected into the lesions of CD45.1 mice. Two days after injection, the recipient mice were euthanized, and GzmB expression in CD8^+^ T cells in the ear and dLNs was analyzed by flow cytometry directly ex vivo. For the RAG^–/–^ mouse model, splenocytes from WT or Blimp-1^cKO^ mice were collected, RBCs were lysed with ACK lysing buffer (Lonza), and CD8^+^ T cells were purified using a magnetic bead separation kit (Miltenyi Biotec). CD8^+^ T cells (3 × 10^6^ cells) were transferred intravenously into RAG^–/–^ mice that were subsequently infected with *L*. *braziliensis*. Mice reconstituted with CD8^+^ T cells received 4 injections of 250 μg anti-CD4 (clone GK1.5, Bio X Cell, catalog BE0003-1) within the first 2 weeks.

### In vivo treatment.

FTY720 (Cayman Chemical) was administered intraperitoneally (3 mg/kg) daily for 10 days before euthanasia, starting at two days post-infection. To induce Cre expression in the ODD^Cre^/ERT2 mice, tamoxifen (MilliporeSigma) was dissolved in corn oil and administered intraperitoneally (6.7 mg/kg) for 7 days following infection. Pimonidazole (Hypoxyprobe) was administered intraperitoneally (9 mg/mL, 200 μL) 1 hour before euthanasia. For neutrophil depletion, mice received every 3 days injections of 250 μg anti-Ly6G (clone 1A8, Bio X Cell, catalog BE0075-1) within the first 2 weeks.

### Tissue single-cell suspension preparation.

Infected ears were harvested, the dorsal and ventral layers of the ear were separated, and the ears were incubated in RPMI 1640 (Gibco, Thermo Fisher Scientific) with 250 μg/mL Liberase TL (Roche Diagnostics) for 60–90 minutes in a shaker at 37°C and 5% CO_2_. Ears were dissociated using a cell strainer (40 μm, BD Pharmingen), and an aliquot of the cell suspension was used for parasite titration. dLNs were homogenized using a cell strainer (40 μm, BD Pharmingen) to obtain single-cell suspensions.

### Parasite titration.

The parasite burden in the ears was quantified as described previously (117). Briefly, the homogenate was serially diluted and incubated at 26°C. The number of viable parasites was calculated from the highest dilution at which parasites were observed after 7 days.

### In vitro stimulation.

dLN single-cell suspensions from infected C57BL/6 mice were cultured with 5 μg/mL plate-bound anti-CD3 (clone 145-2C11, Invitrogen, Thermo Fisher Scientific) and 0.5 μg/mL soluble anti-CD28 (clone 37.51, Invitrogen, Thermo Fisher Scientific) at 37°C and 5% CO_2_ for 48 hours in RPMI 1640 containing 100 Units of penicillin and 0.1 mg/mL streptomycin (MilliporeSigma), 2 mM l-glutamine (Thermo Fisher Scientific), and 10% FBS (MilliporeSigma). Cells were cultured in 1 mM DMOG (MilliporeSigma) dissolved in DMSO or an equivalent volume of DMSO (vehicle) for 48 hours. For mRNA isolation, dLN single-cell suspensions from infected C57BL/6 mice were obtained, and CD8^+^ T cells were purified using a magnetic bead separation kit (Miltenyi Biotec) and cultured as described above. For in vitro hypoxia experiments, cells were subjected to either normoxic conditions (standard incubator) or 1% oxygen (Baker Ruskinn InvivO_2_ 400) in an incubator equipped to replace oxygen with nitrogen.

### Flow cytometric analysis.

Before surface and intracellular staining, cell suspensions were stained with a LIVE/DEAD Fixable Aqua Dead Cell Stain kit (Thermo Fisher Scientific), according to the manufacturer’s instructions. Analysis was performed using FlowJo Software (Tree Star), and gates were drawn on the basis of the fluorescence minus one (FMO) control. The following antibodies were used: CD45 (clone 30-F11, catalog 47-0451-82), GzmB (clone GB11, catalog GRB05 or GRB04), CD11b (clone M1/70, catalog 48-0112-82), IFN-γ (clone XMG1.2, catalog 25-7311-82), IL-10 (clone JES5-16E3, catalog 12-7101-82), Tim3 (clone RMT3-23, catalog 416-5870-82), and CD4 (clone RM4-5, catalog 17-0042-83 or 47-0042-82) (all from Invitrogen, Thermo Fisher Scientific); CD3 (clone 17A2, catalog 741319) and CD44 (clone IM7, catalog 751414) (both from BD Biosciences); CD45.2 (clone 104, catalog 109813), CD45.1 (clone A20, catalog 110705), CD90 (clone 53-2, catalog 140306), CD8β (clone YTS156.7.7, catalog 126610), Ly6G (clone 1A8, catalog 127614), Lag3 (clone C9B7W, catalog 125226), and PD-1 (clone 29F.1A12, catalog 135224) (all from BioLegend). The stained cells were run on a BD FACSymphony A3 or a FACSCanto II (BD Biosciences).

### Quantification of Prdm1 mRNA by quantitative reverse transcription PCR.

RNA extraction of in vitro–stimulated CD8^+^ T cells was performed using the Nulceospin RNA Mini kit (Macherery-Magel), and extracted RNA was then used to prepare cDNA using iScript Reverse Transcription Supermix for reverse transcription quantitative PCR (RT-qPCR) (Bio-Rad) on an ABS Proflex Thermal cycler (Thermo Fisher Scientific). qRT-PCR was carried out on a C1000 RT-PCR system using iTaq Universal SYBR Green Supermix (all from Bio-Rad) and primers targeting *Prdm1* (forward, 5′-TTCTCTTGGAAAAACGTGTG G-3′ and reverse, 5′-GGAGCCGGAGCTAGACTTG-3′). The qRT-PCR results were normalized to *Actb* (forward, 5′-CGCTGTATTCCCCTCCATCG-3′ and reverse, 5′-CCAGTTGGTAACAATGCCATGT-3′). All reactions were carried out in duplicate, and data are represented as the FC over the average expression of normoxic or vehicle-treated cells.

### Histology.

Two weeks after infection, the mice were euthanized, and infected skin (lesions) and contralateral (naive skin) ears were collected and immediately immersed in 2% paraformaldehyde (Thermo Fisher Scientific) at room temperature. Samples were rinsed and immersed in PBS (Gibco, Thermo Fisher Scientific) for 2 hours, followed by cryoprotection in 30% sucrose. Samples were preserved in OCT (Thermo Fisher Scientific), and serial sections of 15 μm were obtained on a Tissue-Tek Cryo_3_ Flex cryostat (Sakura Finetek). Sections were collected onto Superfrost Plus microscope slides (Thermo Fisher Scientific), air-dried, and stored at –80°C.

### Immunofluorescence, image acquisition, and analysis.

Briefly, tissue sections were blocked and incubated with rabbit anti–pimonidazole antisera (Hypoxyprobe, catalog Pab2627), followed by incubation with rat anti–mouse Ly6G (clone 1A8, BioLegend, catalog 127604). Finally, sections were incubated separately with Alexa Fluor 568 goat anti-rabbit (Invitrogen, Thermo Fisher Scientific, catalog A11011) and Alexa Fluor 488 goat anti-rat (Invitrogen, Thermo Fisher Scientific, catalog A11008). The sections were counterstained with the nuclear probe DAPI (Biotechne-Tocris), covered with ProLong Gold Antifade Mountant (Invitrogen, Thermo Fisher Scientific) and kept protected from the light until imaging. Representative images were acquired with ×10 and ×20 objectives on an Olympus FV3000 confocal microscope (Olympus Corporation). The color of the different structures was digitally inverted to better match the data presented in this work. Using ImageJ software, version 1.53 (NIH), 27 regions of interest (ROIs) measuring 600 × 40 μm were created. From the low signal edge to the other, the pixel intensity values of each ROI were obtained.

### Transcriptional profiling of purified mouse CD8^+^ T cells.

C57BL/6 mice were infected with *L*. *major,* and 5 weeks after infection, the mice were euthanized; cells from the infected ear and dLN cells were purified and stained for flow cytometric analysis. Antigen-experienced CD8^+^ T cells were purified using the FACSAria cell sorter (gating strategy: live, singlets, CD90^+^, CD8β^+^CD44^hi^), and RNA was extracted with the RNeasy Plus Mini Kit (QIAGEN) according to the manufacturer’s instructions and used to prepare Poly(A)+-enriched cDNA libraries with the Illumina TruSeq Stranded mRNA library prep workflow. Ribo-Zero Gold rRNA depletion (Illumina) removed the ribosomal content. Quality assessment and quantification of RNA preparations and libraries were performed using the Agilent 4200 TapeStation and the Qubit 3, respectively. Samples were sequenced on an Illumina NextSeq 500 to produce 75 bp single-end reads. The raw reads were mapped to the mouse reference transcriptome (Ensembl; *Mus musculus* version 108) using Kallisto, version 0.46.0, and MultiQC, version 1.9, was used to check the quality of the alignment.

All subsequent analyses were conducted using the statistical computing environment R, version 4.1.0, RStudio, version 1.4.1717, and Bioconductor, version 3.13. Normalized gene expression matrix available for download as a text file in the NCBI Gene Expression Omnibus (GEO) database (GSE193005). Transcript quantification data were summarized to genes using the BiomaRt and tximport package and normalized using the trimmed mean of M-values (TMM) method in edgeR. Genes with less than 1 counts per million (cpm) in at least 3 samples were filtered out. Normalized filtered data were variance stabilized using the voom function in limma, and DEG analysis was performed with linear modeling using limma after correcting for multiple testing with Benjamini-Hochberg FDR correction. The list of 7 genes encoding for transcription factors associated with effector-like T cell functions was gathered from (58–60). Hypoxic enrichment per RNA-Seq sample was calculated with single-sample GSEA using the GSVA R package. The 5 hypoxia-related signatures used to estimate those levels were downloaded from Harris et al. 2002 (62), Hallmark systematic name (sn) no. M5891, Biocarta sn no. M13324, Biocarta sn no. M14863, and Reactome sn no. M641. The gene expression signatures of “stem-like” (CD101^neg^Tim3^neg^), “transitory” (CD101^neg^Tim3^pos^), and “exhausted” (CD101^pos^Tim3^pos^) PD-1^pos^ CD8^+^ T cells were built from the normalized counts data set published elsewhere (118). Here, the normalized counts data were filtered for genes expressing more than 1 cpm in at least 3 samples and normalized with the limma R package. Individual differential gene expression was performed between “stem-like” versus naive, “transitory” versus naive, and “exhausted” versus naive CD8^+^ T cells using the edgeR R package. The lists of the top 100 DEGs from each comparison were used to build the state-of-exhaustion signatures. Single-sample GSEA using the GSVA R package was used to calculate state-of-exhaustion enrichment scores of CD8^+^ T cells isolated from dLNs and lesions of *L*. *major*–infected mice.

### Hypoxia-related transcriptional analyses on blood and lesion biopsies from healthy individuals and patients with cutaneous leishmaniasis.

Data in Figure 6 are derived from published transcriptional profiling (54, 85). The analysis carried out for this current study was performed from the filtered, normalized gene expression matrix available for download as a text file in the NCBI GEO database (GSE162760 and GSE214397). All subsequent analyses were conducted similarly to the analysis used for the purified mouse CD8^+^ T cells RNA-Seq data set. Hypoxia scores for the human RNA-Seq data set were calculated using the Harris et al. gene signature (62). Unsupervised hierarchical clustering to classify lesion samples according to their “hypoxic scores” was performed with maximum distances and Ward’s D2 minimum variance. PERMANOVA statistical testing was performed with the vegan R package. GSEA was performed using Broad Institute software, version 4.3.2. Gene Ontology analyses were carried out using DAVID Bioinformatics Resources (2021 Update) from the National Institute of Allergy and Infectious Diseases (NIAID), NIH and biological process terms, clustering gene sets by annotation similarity. Microenvironment cell populations (MCP) counter and immundeconv R packages were combined to estimate neutrophil abundances from the unstructured RNA-Seq data set. Data visualization was mostly performed in GraphPad Prism, version 10 (GraphPad Software) and in R programming language using the tidyverse and gplots tools.

### Statistics.

For differences between 2 groups, statistical significance was determined using an unpaired 1- or 2-tailed Student’s *t* test or a paired Student’s *t* test. For multiple comparisons, 1-way ANOVA was performed. The specific transcriptional analysis section describes detailed statistical analysis for RNA-Seq. Differences were considered significant when *P* was less than or equal to 0.05. Data are presented as the mean ± SEM.

### Study approval.

This study was conducted according to the recommendations in the NIH *Guide for the Care and Use of Laboratory Animals* (National Academies Press, 2011). The IACUCs of The Ohio State University and the University of Pennsylvania approved the study protocol.

### Data availability.

The complete RNA-Seq data analysis, R code, and file inputs and outputs used to perform the transcriptional analysis presented in this work are available in the GitHub repository “Hypoxia_FON” (https://github.com/camilafarias112/Hypoxia_FON). For all data values for all graphs, see the Supporting Data Values file.

## Author contributions

EAF, CFA, RAS, EDSH, KM, LAS, WZ, CIDO, GDW, PLC, and FON designed the research studies. EAF, CFA, RAS, EDSH, KM, LAS, WZ, FON, AV, AY, and PLC conducted experiments. EAF, CFA, KAM, WZ, and FON analyzed and interpreted the data. EAF, CFA, and FON wrote the manuscript. FON supervised the study.

## Supplementary Material

Supplemental data

Supplemental table 1

Supplemental table 2

Supplemental table 3

Supplemental table 4

Supplemental table 5

Supplemental table 6

Supporting data values

## Version 1 06/04/2024

In-Press Preview

## Version 2 07/15/2024

Electronic publication
