# Supplementary material for: Neutrophil-mediated hypoxia drives pathogenic CD8+ T cell responses in cutaneous leishmaniasis
Source: J Clin Invest. 2024 Jun 4;134(14):e177992. doi: 10.1172/JCI177992 (PMC11245163; doi:10.1172/JCI177992)
Supplement: Supplemental data [file jci-134-177992-s142.pdf]

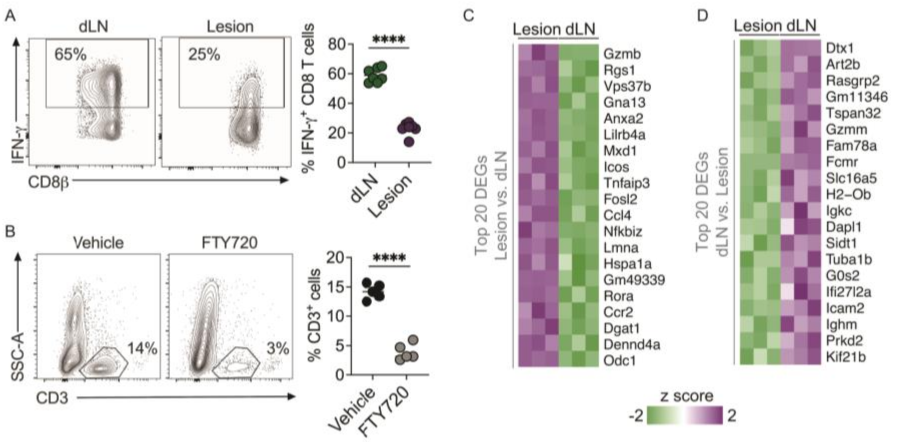

Supplemental Figure 1. Differences between CD8 T cells in lesions and dLNs.

(**A**) IFN-γ expression in CD8 T cells from dLNs and lesions from C57BL/6 mice in week two post-infection with *L. major*. Representative data from more than three experiments with at least four mice per experiment. (**B**) Frequency of T cells in lesions from *L. major* infected C57BL/6 mice treated with FTY720 or vehicle daily 12 days post-infection for 10 days. Data representative from two independent experiments with five mice per group. (**C**-**D**) C57BL/6 mice were infected with *L. major*, and at the peak of the lesion, antigen-experienced ($CD44^{high}$) CD8 T cells were purified by flow cytometry cell sorting and used for RNA-seq. Heat map of top 20 differentially expressed genes overexpressed in CD8 T cells (**C**) from lesions compared to dLN, and (**D**) from dLN compared to lesions. ****$P \leq 0.0001$, by 2-tailed Student's *t*-test (**A** and **B**).

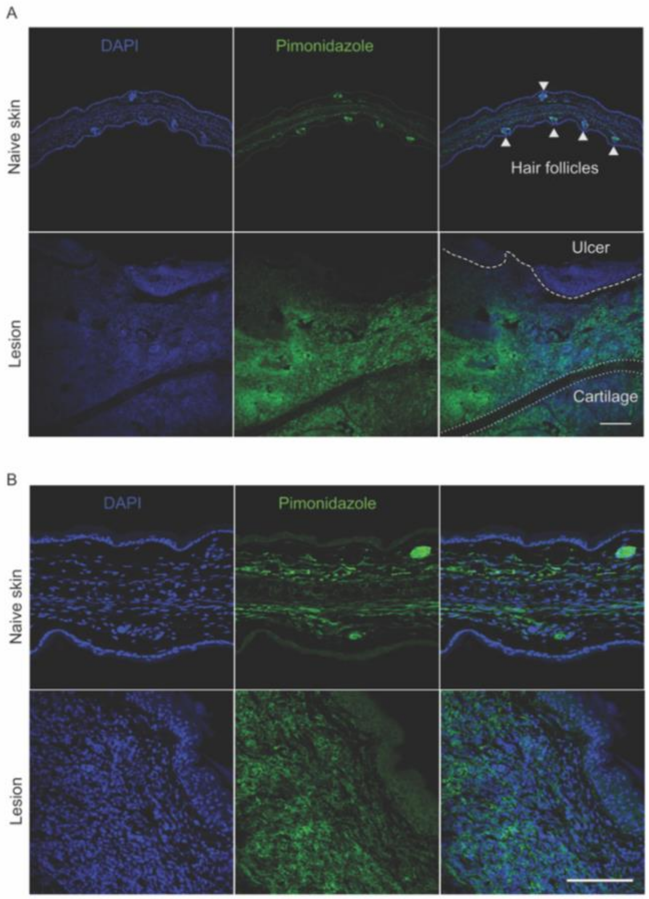

Supplemental Figure 2. Pimonidazole expression in *Leishmania*-infected skin.

C57BL/6 mice infected with *L. major* received pimonidazole one hour before euthanasia at two weeks. (**A**) Individual images from confocal microscopy (Figure 2A). Nuclear (DAPI) staining (blue) and pimonidazole (green) in contralateral ears (naïve skin) and infected ears (lesions) for two weeks. Scale bar = 200 μm. (**B**) Individual images from confocal microscopy (Figure 2B). Nuclear (DAPI) staining (blue) and pimonidazole (green) in contralateral ears (naïve skin) and infected ears (lesions) for two weeks. Scale bar = 100 μm.

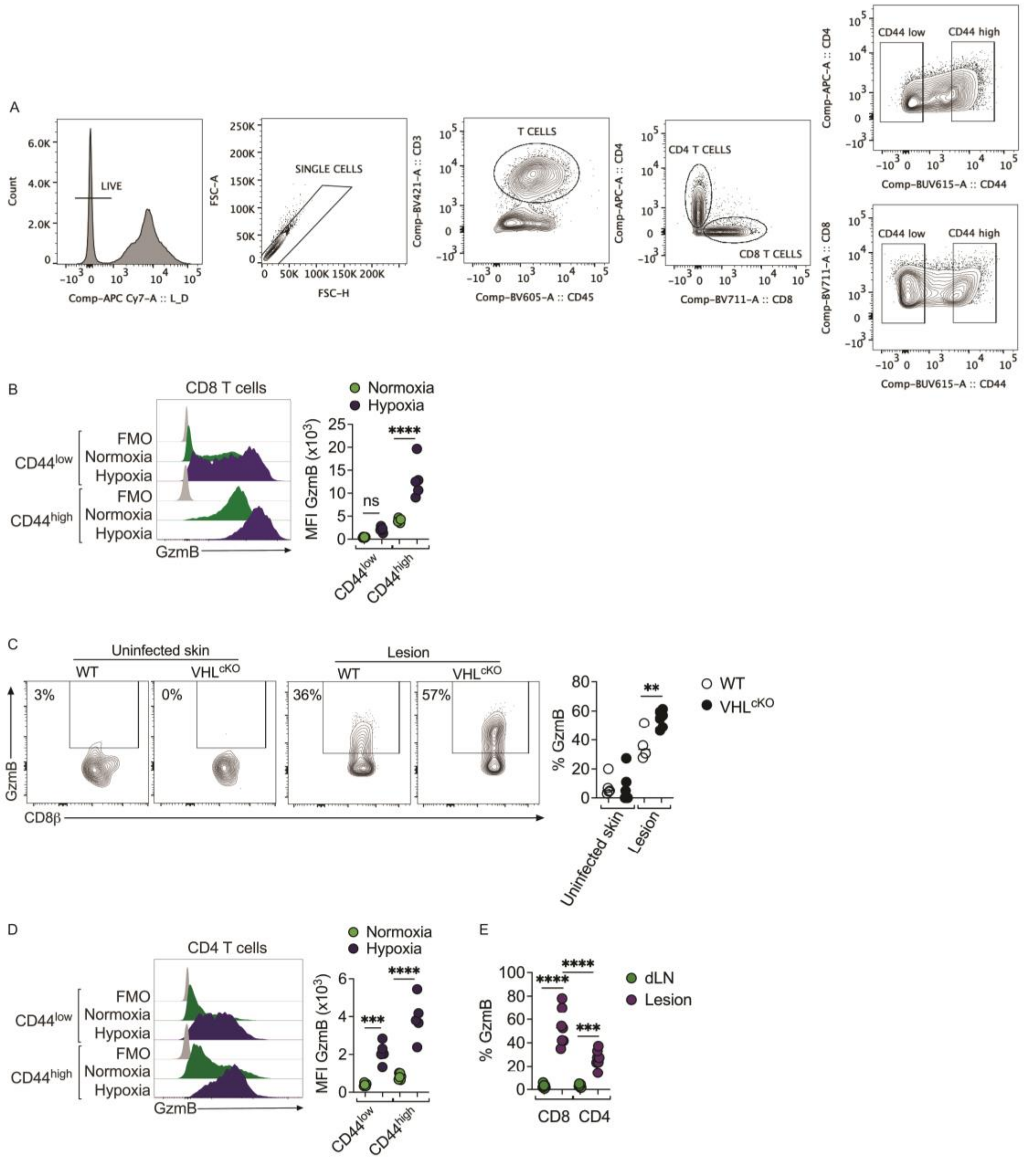

Supplemental Figure 3. Hypoxia induces GzmB expression in CD8 and CD4 T cells.

(**A**) Gating strategy for experiments in Figure 2D-F and Supplemental Figure 3B and D. (**B**) Granzyme B (GzmB) expression in naïve (CD44^{low}) or antigen-experienced (CD44^{high}) CD8 T cells from dLNs of C57BL/6 mice infected with *L. major* stimulated with anti-CD3 and anti-CD28 and cultured under normoxia (21% O_2) or hypoxia (1% O_2). (**C**) GzmB frequency in CD8 T cells in lesions of WT or VHL^{cKO} mice infected with *L. major* for two weeks. (**D**) GzmB expression in naïve (CD44^{low}) or antigen-experienced (CD44^{high}) CD4 T cells from dLNs of C57BL/6 mice infected with *L. major* stimulated with anti-CD3 and anti-CD28 and cultured under normoxia (21% O_2) or hypoxia (1% O_2). (**E**) GzmB expression in CD4 T cells from dLNs and lesions from C57BL/6 mice in week two post-infection with *L. major*. (**A**, **B,** and **D**) Representative data from more than three experiments with at least four mice per experiment. (**C**) Data from two independent experiments combined. (**E**) Representative data from three experiments with at least five mice per experiment. **$P \leq 0.01$, ***$P \leq 0.001$ and ****$P \leq 0.0001$, by one-way ANOVA. ns = non-significant FMO = Fluorescence Minus One.

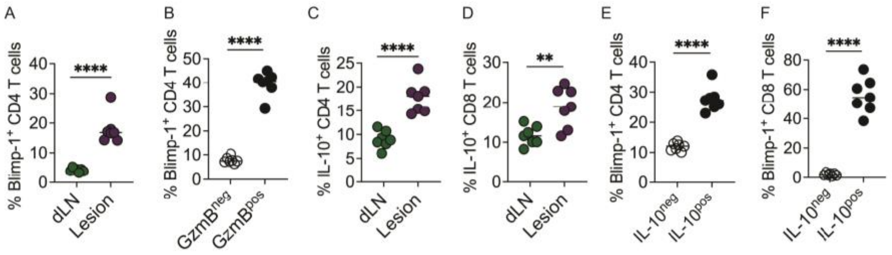

Supplemental Figure 4. The impact of Blimp-1 expression in CD4 and CD8 T cells.

Blimp-1 YFP reporter mice were infected with *L. major* for one week. (**A**) Blimp-1 YFP reporter mice expression in CD4 T cells from dLNs and lesions. (**B**) Blimp-1 YFP expression in Granzyme B (GzmB)^{pos} or GzmB^{neg} CD4 T cells. (**C**-**D**) IL-10 expression in (**C**) CD4 and (**D**) CD8 T cells from dLNs and lesions. (**E**-**F**) Blimp-1 YFP expression in IL-10^{pos} or IL-10^{neg} (**E**) CD4 T cells and (**F**) CD8 T cells. Representative data from three independent experiments with 3-8 mice per experiment. **P ≤ 0.01, and ***P ≤ 0.001, by 2-tailed Student's *t*-test (**A**, **C**, and **D**) and paired *t*-test (**B**, **E**, and **F**). ns = non-significant.

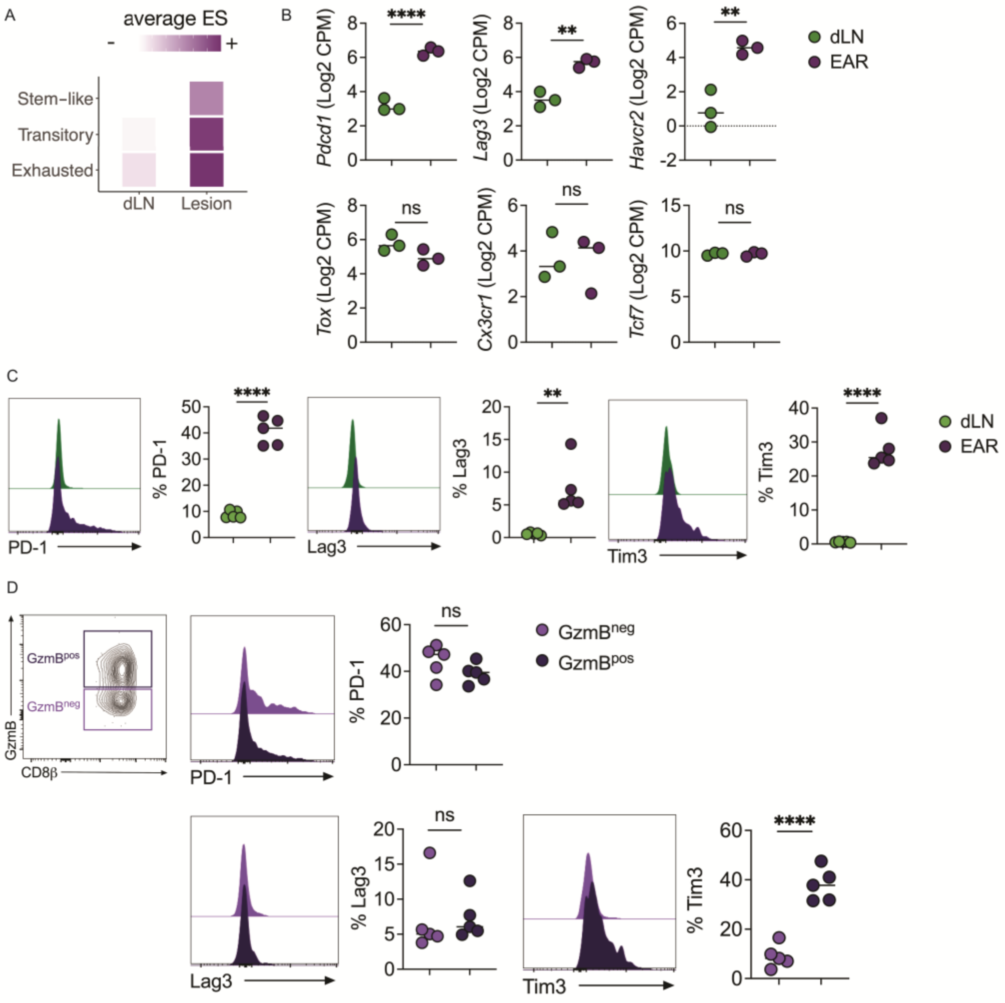

Supplemental Figure 5. CD8 T cells from lesions express markers of terminal exhaustion.

C57BL/6 mice were infected with *L. major*, and at the peak of the lesion, antigen-experienced (CD44^{high}) CD8 T cells were purified by flow cytometry cell sorting and used for RNA-seq. (**A**) Enrichment score (ES) of 'Stem-like,' 'Transitory,' and 'Exhausted' (states-of-exhaustion) gene expression signatures in CD8 T cells. (**B**) Gene expression of exhaustion markers in CD8 T cells from dLN and lesions. (**C**) PD-1, Lag3, and Tim3 expression in CD8 T cells from dLNs and lesions from C57BL/6 mice in week two post-infection with *L. major*. (**D**) PD-1, Lag3, and Tim3 expression in granzyme B (GzmB) positive and negative CD8 T cells from lesions. (**C**-**D**) Representative data from two experiments with five mice per experiment. **P ≤ 0.01, and ****P ≤ 0.0001, by 2-tailed Student's *t*-test (**B**-**D**). ns = non-significant.

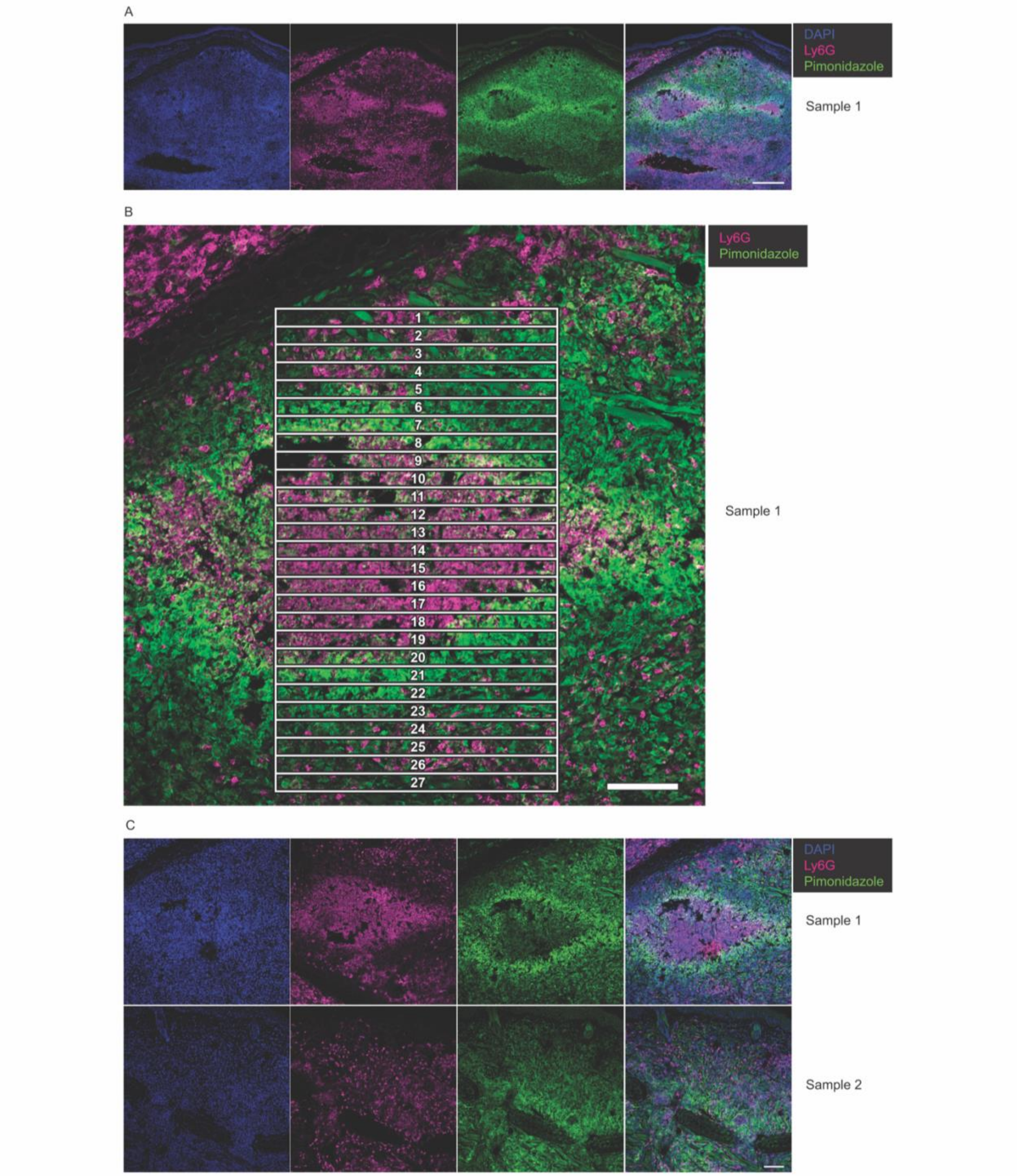

Supplemental Figure 6. Neutrophil and pimonidazole expression in *Leishmania*-infected skin.

C57BL/6 mice infected with *L. major* received pimonidazole one hour before euthanasia at two weeks. (**A**) Individual images from confocal microscopy (Figure 5C). Nuclear (DAPI) staining (blue), Ly6G (pink), and pimonidazole (green) in the skin infected for two weeks. Scale bar = 200 μm. (**B**) The confocal microscopy image was divided into 27 regions to measure the pixel intensity of Pimonidazole (green) and Ly6G (pink) shown in Figure 5D. Scale bar = 100 μm. (**C**) Individual images from confocal microscopy (Figure 5E). Nuclear (DAPI) staining (blue), Ly6G (pink), and pimonidazole (green) in the skin infected for two weeks. Scale bar = 100 μm.
